# Distribution of GFAP in Squamata: Extended Immunonegative Areas, Astrocytes, High Diversity, and Their Bearing on Evolution

**DOI:** 10.3389/fnana.2020.00049

**Published:** 2020-08-14

**Authors:** Dávid Lõrincz, Mihály Kálmán

**Affiliations:** ^1^Faculty of Veterinary Science, University of Veterinary Medicine, Budapest, Hungary; ^2^Department of Anatomy, Histology and Embryology, Semmelweis University, Budapest, Hungary

**Keywords:** brain evolution, glial fibrillary acidic protein (GFAP), lizards, snakes, turtles

## Abstract

Squamata is one of the richest and most diverse extant groups. The present study investigates the glial fibrillary acidic protein (GFAP)-immunopositive elements of five lizard and three snake species; each represents a different family. The study continues our former studies on bird, turtle, and caiman brains. Although several studies have been published on lizards, they usually only investigated one species. Almost no data are available on snakes. The animals were transcardially perfused. Immunoperoxidase reactions were performed with a mouse monoclonal anti-GFAP (Novocastra). The original radial ependymoglia is enmeshed by secondary, non-radial processes almost beyond recognition in several brain areas like in other reptiles. Astrocytes occur but only as complementary elements like in caiman but unlike in turtles, where astrocytes are absent. In most species, extended areas are free of GFAP—a meaningful difference from other reptiles. The predominance of astrocytes and the presence of areas free of GFAP immunopositivity are characteristic of birds and mammals; therefore, they must be apomorphic features of Squamata, which appeared independently from the evolution of avian glia. However, these features show a high diversity; in some lizards, they are even absent. There was no principal difference between the glial structures of snakes and lizards. In conclusion, the glial structure of Squamata seems to be the most apomorphic one among reptiles. The high diversity suggests that its evolution is still intense. The comparison of identical brain areas with different GFAP contents in different species may promote understanding the role of GFAP in neuronal networks. Our findings are in accordance with the supposal based on our previous studies that the GFAP-free areas expand during evolution.

## Introduction

Squamata is one of the richest and most diverse extant groups of Reptilia (Zug et al., 2001). The present study aims to characterize the glial fibrillary acidic protein (GFAP)-immunopositive elements of five lizard and three snake species, which represent different families of Squamata (Table 1). GFAP is the main intermediate filament and immunohistochemical marker of astroglia (Bignami et al., 1980). This study continues our former ones on the distribution of GFAP in different vertebrates including chicken (Kálmán et al., 1993, 1998), turtles (Kálmán et al., 1994, 1997), and caiman (Kálmán and Pritz, 2001).

**TABLE 1:** Species studied with their taxonomical positions.

Classis	Ordo (Subclass)	Subordo	Familia	Species and the number of animals
Reptilia	Squamata (Lepidosauria)	Lacertomorpha	Lacertidae	Moroccan eyed lizard – *Timon tangitanus* (BOULENGER, 1889) – 2
		Iguania	Agamidae	Bearded dragon *– Pogona vitticeps* (AHL, 1926) *–* 4
			Chamaeleonidae	Veiled chameleon *– Chamaeleo calyptratus* (DUMÉRIL and DUMÉRIL, 1851) *–* 3
		Anguimorpha	Varanidae	Savannah monitor (*Varanus exanthematicus* Bosc, 1792*) –* 1
		Gekkota	Eublepharidae	Leopard gecko *– Eublepharis macularius* (BLYTH, 1854) *–* 4
		Serpentes	Boidae	Columbian rainbow boa *– Epicrates cenchria maura* (LINNAEUS, 1758) *–* 2
			Pythonidae	Ball python *– Python regius* (SHAW, 1802) *–* 2
			Colubridae	Corn snake *– Pantherophis guttatus* (LINNAEUS, 1766) *–* 3
	Crocodilia (Archosauria)		Alligatoridae	Cuvier’s dwarf caiman *Paleosuchus palpebrosus* (CUVIER, 1807) *–* 2
	Testudines (undefined*)	Cryptodira	Testudinidae	Hermann’s tortoise *– Testudo hermanni boettgeri* (MOJSISOVICS, 1889) *–* 1
			Emydidae	Red-eared slider *– Trachemys scripta elegans* (WIED 1838) *–* 2
			Geoemydidae	Chinese stripe-necked turtle *– Mauremys sinensis* (GRAY, 1834) *–* 2
		Pleurodira	Pelomedusidae	African helmeted turtle *– Pelomedusa subrufa* (BONNATERRE, 1789) *–* 2

*See section “Discussion,” end of “Diversity and Evolution.”

These studies demonstrated that the predominance of astrocytes and the appearance of large brain areas poor in GFAP immunopositivity are characteristic of birds and mammals but not found in either turtles or caiman, in which almost evenly dense, thin, elongated astroglial processes (the “tanycytes” of Horstmann, 1954) are predominant (for a review, see Kálmán, 2002). The present study continues to investigate these phenomena of astroglial evolution in lizards and snakes, which are lepidosaurs in contrast to birds and caiman, which belong to archosaurs. Although several studies targeted lizards (Dahl et al., 1985; Bodega et al., 1990; Monzon-Mayor et al., 1990; Yanes et al., 1990; Lazzari and Franceschini, 2001, 2005a,b), but the majority of the studies only investigated a single species, except for Ahboucha et al. (2003) who compared three lizard species. Data on snakes are almost completely missing except for one comment by Onteniente et al. (1983). Investigation of several species provided us a possibility to recognize an interfamilial diversity of glial structures.

Considering that our former turtle and caiman studies were also based on single species, another caiman species and four turtle species (Table 1) were also investigated in parallel including a representative of Pleurodira turtles, which taxon has not been studied by either us or other groups. The reason of these latter studies was to check whether the glial architecture of this group shows similar diversities that were found between Squamata.

Presentation of every anatomical detail of all of the brains studied would extend beyond the limits of this paper; therefore, only typical details are shown, and similar areas of different species are demonstrated with one representative figure. Turtles and caiman are only described in brief regarding the former publications.

## Materials and Methods

### Animals, Fixation and Sectioning

The animals (Table 1) were obtained from breeders. They were sublethally overanesthetized with Nembutal (Ceva Gmbh, Düsseldorf, Germany, 20 mg/kg) and transcardially perfused with paraformaldehyde (Merck, Darmstadt, Germany) solution, 4% in phosphate buffered saline (Sigma-Aldrich, St. Louis, MO, United States). Following 2 days postfixation, the brains were embedded into agarose and a series of coronal sections (50–70 μm) were cut by Vibratome (Intracel, Shepreth Royston Herts, United Kingdom).

The experiments were performed in accordance with the Committee on the Care and Use of Laboratory Animals of the Council on Animal Care at the Semmelweis University of Budapest, Hungary (22.1/3491/003/2008), the permission of Hungarian authorities (KA-1928, dated from May 31, 1916), and the European Union Directive (EU Directive 2010/63/EU).

### Immunohistochemical Procedure

After rinsing overnight in phosphate buffer, the floating sections were pretreated with 3% H_2_O_2_ (for 5 min) to suppress the endogenous peroxidase activity and then incubated in 20% normal goat serum (Vector Labs, Burlingam, United Kingdom, for 1.5 h, at room temperature) to block the non-specific antigen binding. These and the following steps all included a rinse with phosphate buffer between the change of reagents. The anti-GFAP was a Novocastra (Newcastle-upon-Tyne, United Kingdom) monoclonal anti-mouse antibody (Code Nu. ga5, PRID AB 563739). It was diluted 1:100 (final conc. 100 μg/ml) in phosphate buffer containing 0.5% Triton X-100 (Sigma-Aldrich, Düsseldorf, Germany) and applied for 40 h at 4°C. As a secondary antibody biotinylated donkey anti-mouse immunoglobulin was used, and then the sections were incubated with streptavidin-biotinylated horseradish peroxidase complex, both from Vector Labs (Burlingam, United Kingdom), diluted 1:100 in phosphate buffered saline and applied for 1.5 h at room temperature. The final concentration of secondary antibody was 15 μg/ml. The immunocomplex was visualized by diaminobenzidine (DAB, Amersham, United Kingdom) reaction, 0.05% 3–3′-DAB in 0.05 M Tris-HCl buffer (Sigma-Aldrich, St. Louis, MO, United States, pH 7.4) containing 0.01% H_2_O_2_ (Reanal, Budapest, Hungary) at room temperature for 5–10 min, until a brownish color appeared. Every reaction was at least once repeated. Nissl cresyl-violet counterstaining was applied on some sections.

Sections of similar brain areas of different species were incubated together. It helps to rule out that the interspecific differences found are to be attributed to the different qualities of different incubations. As a negative control, the anti-GFAP antibody was omitted from the procedure. In these cases, no structure-bound color product was found. For positive controls, rat brain sections were applied. In some sections, an antigen retrieval with sodium citrate buffer (0.1 M, pH = 6.1, prepared from citric acid monohydrate, Sigma-Aldrich, Düsseldorf, Germany) for 1.5 h on 75°C was performed before the incubation with primary antibodies. The retrieval did not reveal GFAP immunopositivity in the otherwise immunonegative areas only emphasized what was visible without retrieval.

The sections were mounted, dried in air, covered with DePeX (Sigma-Aldrich, St. Louis, MO, United States) and coverslipped. The photomicrographs were taken by a DP50 digital camera mounted on an Olympus BX-51 microscope (both from Olympus Optical Co. Ltd., Tokyo, Japan). Digital images were processed using Photoshop 9.2 software (Adobe Systems, Mountain View, CA, United States) with minimal adjustments for brightness and contrast. Overviews, whole-section photomicrographs were taken with objective × 1.2 or photomontages were prepared from details taken with an objective × 4. To reveal smaller details, objectives ×10, ×20, or × 40 were used. On equivalent areas or details, identical objectives were used. Brain areas were identified based on the works of Smeets et al. (1986) and Ten Donkelaar (1997).

## Results

### Telencephalon and the Anterior Part of the Hypothalamus, Lizards

**FIGURE 1:**
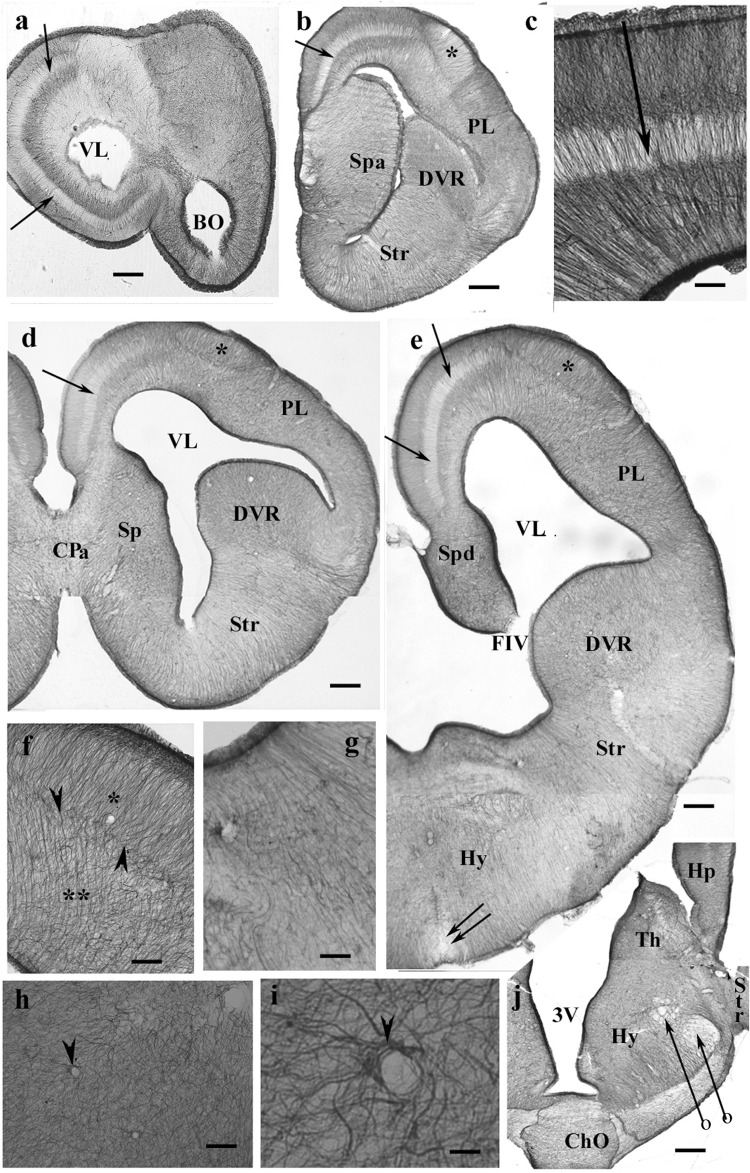
GFAP-immunopositive elements in gecko telencephalon and anterior hypothalamus—a GFAP-rich lizard brain. 3V, third ventricle; BO, olfactory bulb; ChO, optic chiasma; CPa, pallial commissure; DVR, dorsal ventricular ridge; FIV, interventricular foramen; GFAP, glial fibrillary acidic protein; Hp, hippocampus; Hy, hypothalamus; PL, lateral pallium; Sp, septum; Spa, septum, anterior nuclei; Spd, septum, dorsal nuclei; Str, striatum; Th, thalamus; VL, lateral ventricle. **(a)** At the frontal pole of the lateral ventricle. Note the trilaminar structure of pallium (arrow points to the middle light zone here and in the following panels). Scale bar: 240 μm. **(b)** Through the septum (here: its anterior nucleus, note the thickening), in front of the pallial commissure. The radial pattern is masked by processes of other orientations except the separate area around the asterisk (dorsal pallium magnocellular part). Scale bar: 250 μm. **(c)** The trilaminar arrangement enlarged. In the middle layer (arrow), the radial processes are relatively loose; deep and superficial layers: the processes are densely packed. Scale bar: 100 μm. **(d)** At the pallial commissure. The structure is similar to that seen in panels **(a,b)**. Scale bar: 250 μm. **(e)** Through the interventricular foramen; double arrow: optic recess. Scale bar: 250 μm. **(f)** The area marked with asterisk in panels **(b,d,e)** (dorsal pallium, magnocellular part) contains a radial process system different from the more complex glial mesh of the surrounding areas (double asterisk). Note the bordering glial plexus (arrowheads). Scale bar: 50 μm. **(g)** A detail of striatum: radial ependymoglia. Scale bar: 60 μm. **(h)** A detail of the DVR. Complex glial system, no radial orientation is recognizable. Note the perivascular processes (arrowhead). Scale bar: 60 μm. **(i)** Perivascular glia (arrowhead). Scale bar: 12 μm. **(j)** The anterior part of the hypothalamus. The optic chiasm and the medial and lateral forebrain bundles (arrows with circles) are lighter due to the scarce glial elements. Scale bar: 250 μm.

Throughout the gecko telencephalon, the immunopositivity was intense. In the medial and dorsal pallia (Figures 1a–e), radial glia and a layered structure were recognized: a middle zone was conspicuously “light” because the processes were less densely packed (Figure 1c).

In the septum, lateral pallium, and dorsal ventricular ridge (DVR), the glial structure was rather complex (Figures 1d,e), radial arrangement of glial processes was not recognizable, except for a lentiform area of magnocellular cells in the dorsal pallium (Figure 1f). In the striatum (Figure 1g), however, parallel radial ependymoglia was found. In the DVR, the original radial arrangement was interwoven by non-radial processes almost beyond recognition (Figure 1h). The perivascular processes formed wide end-feet on the vessels (Figure 1i). Astrocytes were not found in the telencephalon. The anterior part of the diencephalon was also intensely GFAP-immunopositive (Figure 1j). Its radial glia was enmeshed with non-radial processes almost beyond recognition (for a similar pattern, see Figure 1h).

**FIGURE 2:**
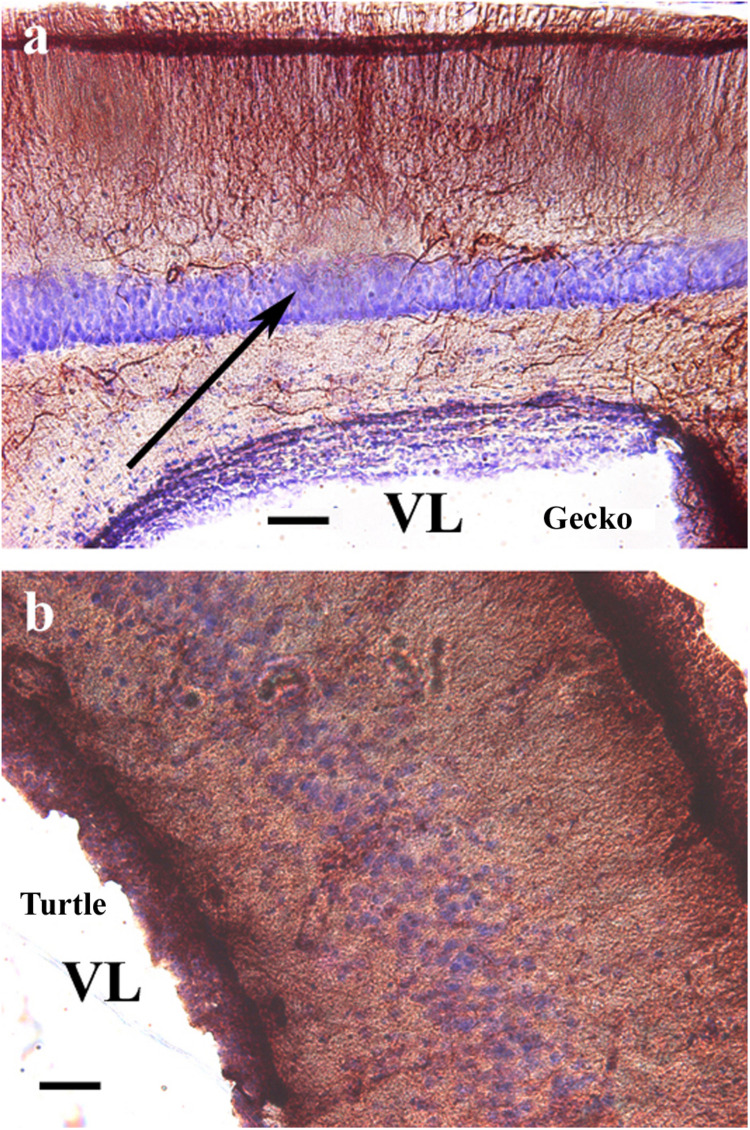
Nissl cresyl violet counterstaining on the dorsomedial pallium. **(a)** Gecko: the light GFAP-poor middle layer (arrow) is full of densely packed neurons. A Nissl cresyl violet counterstaining. **(b)** Turtle *(Mauremys sinensis)* pallium: the glial structure is not trilaminar, and the cell bodies are less densely packed. GFAP, glial fibrillary acidic protein; VL, lateral ventricle. Scale bar: 100 μm.

Counterstaining with cresyl violet according to Nissl demonstrated that the “light” area was occupied by densely packed neurons (Figure 2a). A similar neuronal layer was found in turtles but did not alter the distribution of GFAP. No trilaminar glial structure was found, the pallium was evenly densely rich in GFAP, and the cell bodies were less densely packed (Figure 2b).

**FIGURE 3:**
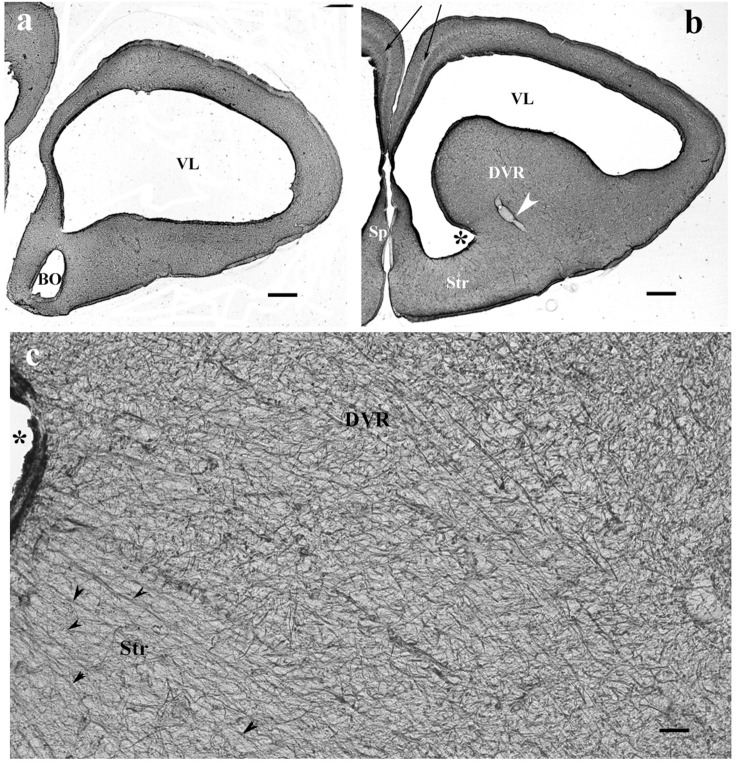
The telencephalon of the monitor lizard is also rich in GFAP immunopositivity. BO, olfactory bulb; DVR, dorsal ventricular ridge; GFAP, glial fibrillary acidic protein; Sp, septum; Str, striatum; VL, lateral ventricle. **(a,b)** Monitor lizard, rostral and cranial sections of the telencephalon both are rich in GFAP. Note the trilaminar pallium (arrows) and the main vessel of DVR (arrowhead). Asterisk labels sulcus ventralis. Scale bar: 800 μm. **(c)** Adjacent parts of DVR and striatum enlarged. The radial arrangement (arrowheads) of glial processes is recognizable in the striatum but hardly in the DVR where it is masked by non-radial processes. Asterisk labels sulcus ventralis, like in panel **(b)**. Scale bar: 30 μm.

In monitor lizard, the telencephalon was also rich in GFAP. A trilaminar pattern of pallium was recognizable (Figures 3a,b) similar to the gecko pallium. The striatum had a radial pattern. A similar pattern was not recognizable in the DVR (Figure 3c). Astrocytes were not seen.

**FIGURE 4:**
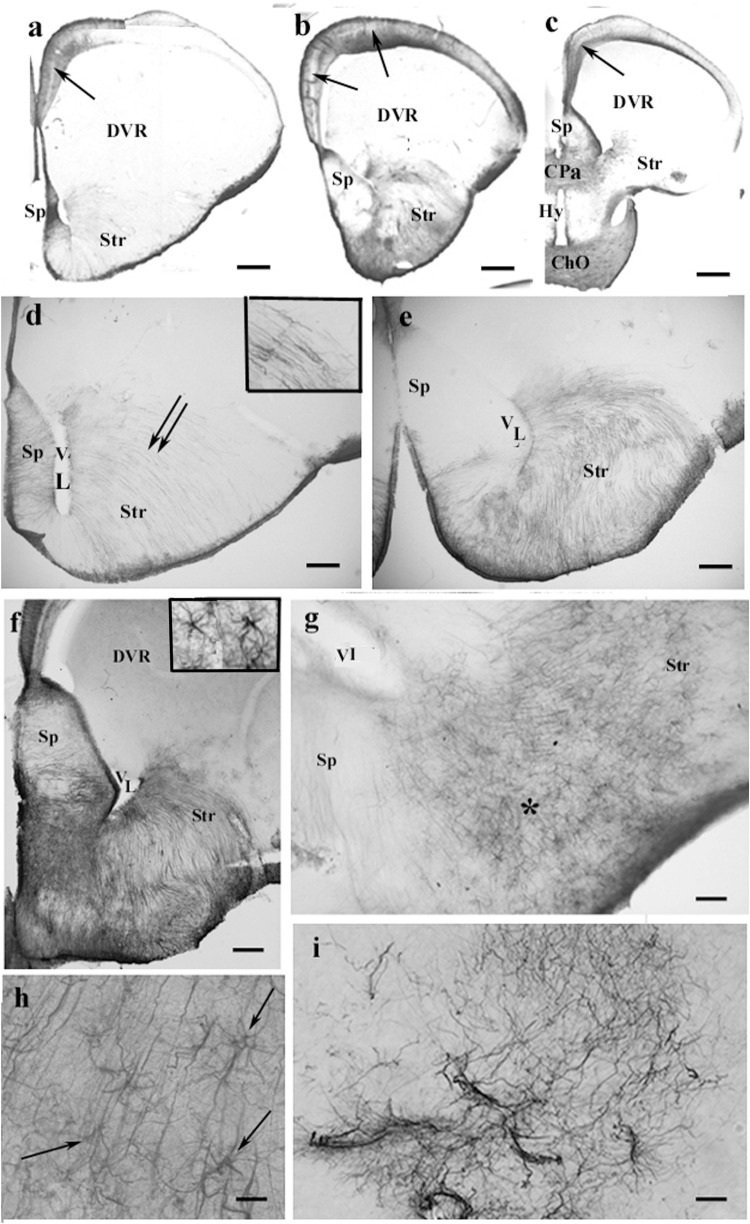
GFAP-immunopositive elements in agama telencephalon—a GFAP-poor brain. ChO, optic chiasma; CPa, pallial commissure; DVR, dorsal ventricular ridge; GFAP, glial fibrillary acidic protein; Hy, hypothalamus; Sp, septum; Str, striatum; VL, lateral ventricle. **(a–c)** Surveys on whole telencephalic hemispheres-in rostrocaudal order. Note the GFAP-rich areas confined but modestly increasing caudalward. The DVR and the anterior part of the hypothalamus remain almost free of GFAP. In the medial and mediodorsal pallia, a trilaminar pattern is found (arrows). In the dorsal pallium, it is not recognizable; the lateral pallium is almost devoid of GFAP. Scale bars: 300, 400, and 600 μm. **(d–f)** The septum and striatum enlarged as areas containing GFAP-immunopositive structures. **(d)** An area enlarged similar to that seen in panel **(a)**. The septum is penetrated by radial glial processes. In the striatum, the radial processes (double arrow, see enlarged in the inset) have been curved due to the uneven thickening of the striatal brain wall. Scale bar: 100 μm. **(e)** An area enlarged, its position is between panels **(a,b)**. The septum is basally penetrated by transversal processes; GFAP immunopositivity also colorizes the glia in the striatum. Scale bar: 100 μm. **(f)** An area enlarged similar to that seen in panel **(b)**. The GFAP immunopositivity is quite intense in the septum and striatum, whereas in the DVR, no GFAP is visible. Inset: septal astrocytes. Scale bar: 100 μm. **(g)** Astrocytes and radial glial processes are intermingled around the asterisk in the nucleus accumbens. See enlarged in panel **(h)**. Scale bar: 40 μm. **(h)** Astrocytes (arrows) enlarged among radial processes in the nucleus accumbens. Scale bar: 20 μm. **(i)** Corresponding to the amygdala “irregular” sinuous processes are found. Scale bar: 50 μm.

In agama, the telencephalon was poor in GFAP-immunopositive structures (Figures 4a–c), which were confined to the medial and mediodorsal pallia, septum, striatum, and amygdala, whereas in the DVR, no GFAP was visible. In the medial and dorsal pallia, a trilaminar pattern was found like in gecko. The GFAP immunopositivity almost avoided the lateral pallium. In the septum and striatum, the territory of GFAP-immunopositive structures increased caudalward (Figures 4d–f). In the striatum, the radial processes were curved as a result of the uneven thickening of the brain wall (Figures 4d,e). Astrocytes occurred in the septum and nucleus accumbens among the radial processes (Figures 4f–h). Corresponding to the amygdala, “irregular” sinuous processes were found (Figure 4i).

**FIGURE 5:**
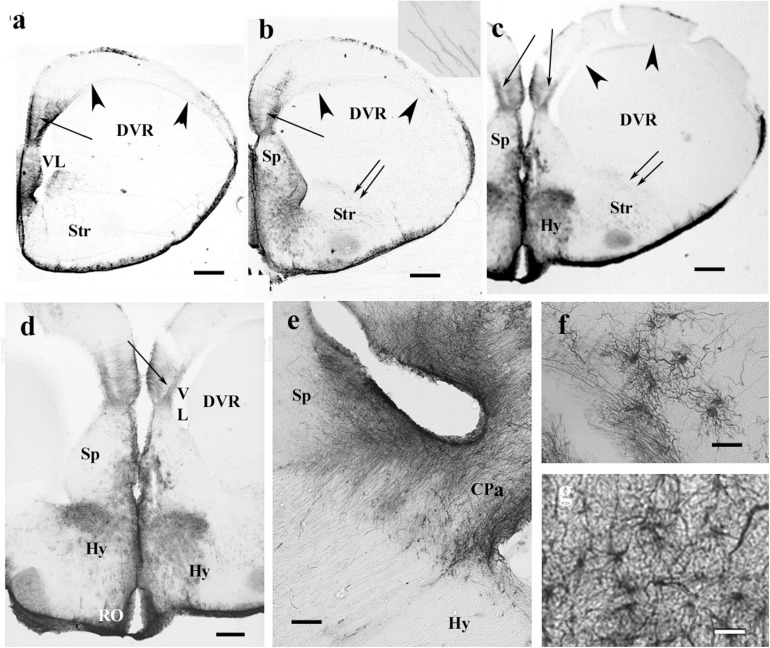
The telencephalon of the chameleon is also poor in GFAP immunopositivity. CPa, pallial commissure; DVR, dorsal ventricular ridge; GFAP, glial fibrillary acidic protein; Hy, hypothalamus; RO, optic recess; Sp, septum; Str, striatum; VL, lateral ventricle. **(a–c)** Rostrocaudal series of sections from the chameleon. The GFAP immunopositivity which visualizes a trilaminar glial pattern is only recognizable in the medial pallium (arrow). The septum is rich in GFAP immunopositivity. The striatum is penetrated by arching long processes (double arrow, see enlarged as inset). The preoptic hypothalamus is covered by GFAP-immunopositive astrocytes. Arrowheads mark the compressed lateral ventricle between DVR and the dorsal pallium. Scale bar: 500 μm. **(d)** Enlarged part of the medial side of the chameleon telencephalon. Note the trilaminar pattern in the medial pallium (arrow) and the GFAP-rich parts of the septum and (preoptic) hypothalamus. Scale bar: 300 μm. **(e)** Pallial commissure of the chameleon. Note the glial processes parallel with the axons. Scale bar: 100 μm. **(f)** Astrocytes from the chameleon septum. Scale bar: 20 μm. **(g)** Astrocytes from the chameleon hypothalamus. Scale bar: 20 μm.

In the chameleon telencephalon, the GFAP-immunopositive structures (Figures 5a–c) only occurred in the medial pallium where the trilaminar glial pattern was recognizable. The DVR was almost free of GFAP. The striatum was penetrated by arching radial processes (Figure 5b). The septum was rich in GFAP immunopositivity as well as the preoptic hypothalamus (Figure 5d). Several GFAP-immunopositive astrocytes were found in both areas (Figures 5f,g). In the pallial commissure, the glial processes run in parallel with the axons (Figure 5e).

**FIGURE 6:**
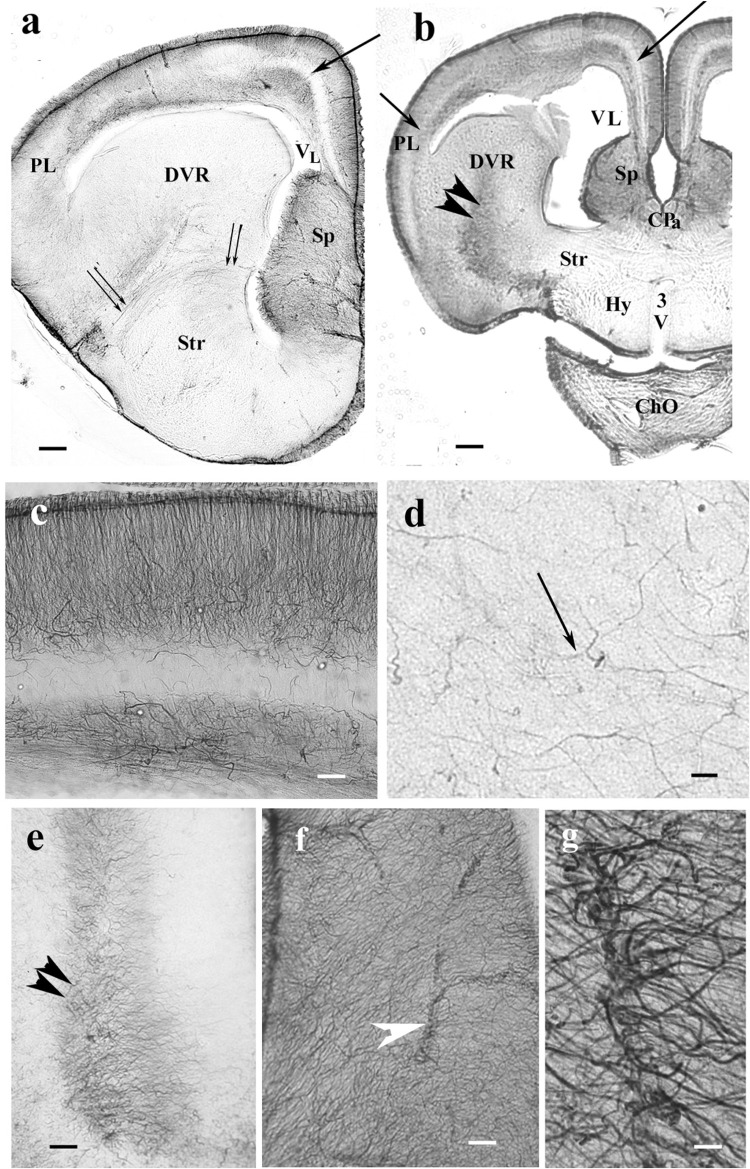
*Timon* telencephalon represents an intermediate extension of GFAP immunopositivity. 3V, third ventricle; ChO, optic chiasma; CPa, pallial commissure; DVR, dorsal ventricular ridge; GFAP, glial fibrillary acidic protein; Hy, hypothalamus; PL, lateral pallium; Sp, septum; Str, striatum; VL, lateral ventricle. **(a,b)** *Timon* telencephalon in front of and through the pallial commissure. The GFAP-poor middle zone (arrows) also appears in the lateral pallium. The septum is rich in GFAP. The radial pattern is masked by non-radial processes. From the striatum, a GFAP-immunopositive zone (double arrowhead) extends to the middle of the DVR, which is otherwise poor of GFAP immunopositivity. The hypothalamus is almost free of GFAP. Below it, the slit is an artifact. Double arrow: arched radial glial processes at the border of the striatum. Scale bars: 200 and 300 μm. **(c)** A part of the pallium enlarged. Note that in this species, the glial processes are not arranged radially in the lowest layer. Scale bar: 100 μm. **(d)** In most of the DVR, the presence of GFAP-immunopositive processes (arrow) is only recognizable under high-power objective. Scale bar: 10 μm. **(e)** The GFAP-rich central zone of the DVR (double arrowhead) enlarged. Scale bar: 100 μm. **(f)** *Timon* septum. The radial process system is enmeshed by other processes and hardly recognizable. Arrowhead: vessel. Scale bar: 70 μm. **(g)** Perivascular end-feet around a vessel enlarged from the previous panel. Scale bar: 10 μm.

The lacertid lizard *Timon* represented an intermediate distribution of GFAP immunopositivity (Figures 6a,b) as compared to the former species. In the pallium, the GFAP-poor middle zone extended into the lateral pallium. In this species, however, the short processes of the lowest zone had irregular courses (Figure 6c). Most of the DVR was poor of GFAP immunopositivity, only high-power objective revealed thin irregular processes (Figure 6d). In the middle of the DVR, there was a GFAP-immunopositive zone (Figure 6e) which continued in the striatum. The preoptic hypothalamus was almost free of GFAP (Figure 6b). In the septum, the radial glial system was enmeshed by non-radial processes and was hardly recognizable (Figure 6f). On vessels, the glial processes terminated with wide, round end-feet (Figure 6g).

### Telencephalon and the Anterior Part of the Hypothalamus, Snakes

**FIGURE 7:**
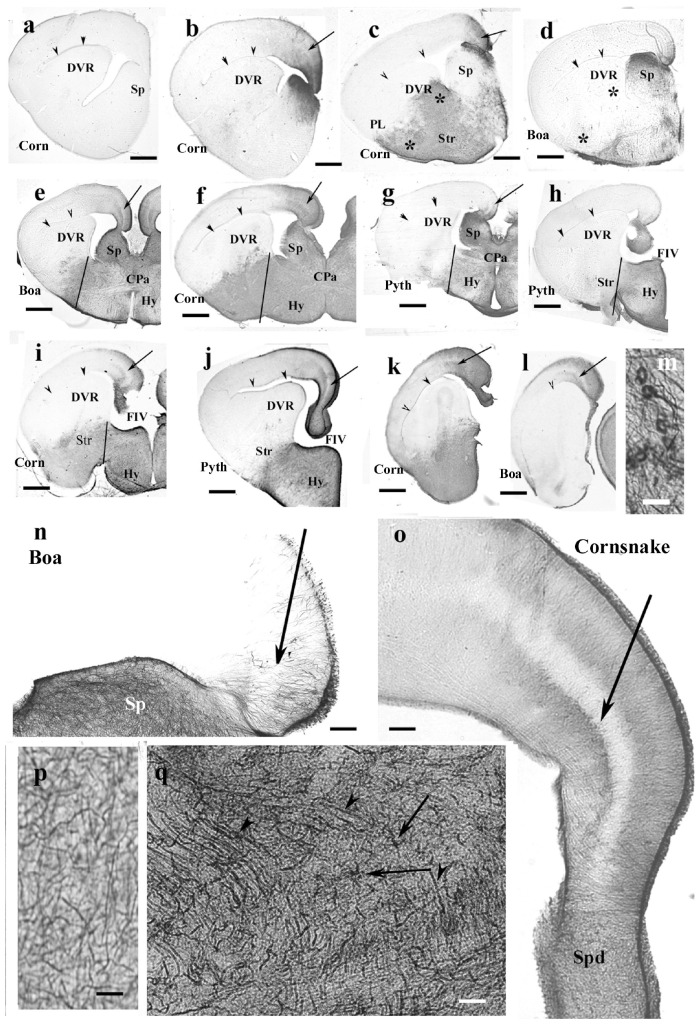
Snakes (Corn, corn snake, Pyth, python)—intermediate distribution of GFAP immunopositivity, only few interspecific differences in the telencephalon. CPa, pallial commissure; DVR, dorsal ventricular ridge; FIV, interventrivular foramen; GFAP, glial fibrillary acidic protein; Hy, hypothalamus; PL, lateral pallium; Sp, septum; Spd, septum, dorsal nuclei; Str, striatum. **(a–l)** Telencephalic sections in rostrocaudal order: the territory of GFAP immunopositivity gradually extends but less in boa and python than in corn snake. Scale bar: 800 μm. **(a)** No GFAP was found in the most rostral sections in either species (here: corn snake). Arrowheads here and in panels **(b–l)** point to the compressed lateral ventricle between the DVR and dorsal pallium. **(b)** GFAP appears first in the mediodorsal pallium and the adjacent part of the septum in either species (here: corn snake). The trilaminar pattern is hardly recognizable [arrow; it points to the similar details in (**c–l**)]. **(c)** The GFAP-immunopositive area extends into the striatum, DVR, lateral pallium, and septum in the corn snake. Arrow here and panels **(d–l)**: the middle layer of the pallium. Asterisks emphasize the difference from boa (**d**). **(d)** The process is less extensive in boa [in **(d)**, note asterisks] and python (not shown). **(e–g)** Sections at the pallial commissure from boa, corn snake, and python, respectively. Lines at the ventral sulcus of the DVR help to compare the extension of GFAP-immunopositive areas. The preoptic hypothalamus is rich in GFAP-immunopositive elements. **(h,i)** Sections at the interventricular foramen. The striatum is only GFAP-immunopositive in corn snake **(i)** but not in python **(h)** and boa (not shown). Lines: see legends of panels **(e–g)**. **(j)** Section from python at the interventricular foramen but caudal from the levels of sections shown in panels **(h,i)**. The preoptic hypothalamus is rich in GFAP immunopositivity but not the striatum. At this level, the trilaminar structure is visible in the medial pallium (arrow). **(k,l)** Sections posterior to the interventricular foramen. Arrowhead points to the artifact rupture of the brain wall where telencephalon was separated from the diencephalon. The level of panel **(k)** (corn snake) is a little rostral to that of panel **(l)** (boa). The trilaminar structure of pallium is visible (arrow). **(m)** Perivascular glial end-feet. Scale bar: 10 μm. **(n)** Enlarged part of the medial pallium (boa) has scarce GFAP-immunopositive processes, the laminar structure is hard to be recognized (arrow). The adjacent part of the septum (Sp) is richly penetrated by GFAP-immunopositive processes. Scale bar: 120 μm. **(o)** The trilaminar structure (arrow: the middle layer) of the medial pallium in corn snake from a caudal section like in panel **(j)**. It ceases in the dorsal septal nuclei. Scale bar: 120 μm. **(p)** Complex system of processes in different directions (corn snake). Radial glia are not recognizable. Similar glial structure is found in the low-power pictures of panels **(c–j)**. Scale bar: 10 μm. **(q)** Astrocytes (arrows) and long processes (arrowheads) in the root of the septum (corn snake). Scale bar: 20 μm.

The corresponding telencephalic sections were very similar in the different species, mainly in boa and python, therefore, they are described and displayed in parallel. In rostrocaudal order, the territory of GFAP immunopositivity gradually extended. The most rostral part of the telencephalon was free of GFAP (Figure 7a). Going caudalward, GFAP immunopositivity firstly appeared in the medial pallium and in the adjacent part of the septum in either species (Figure 7b). The trilaminar pattern seen in lizards was hardly recognizable yet. More caudally, the GFAP immunopositivity extended into the septum and striatum, whereas the DVR remained almost free of it (Figures 7c–j). The preoptic hypothalamus was rich in GFAP (Figures 7e–j). In these areas, no radial pattern was recognizable, only sinuous glial processes without any appreciable system. At the level of the interventricular foramen in the pallium, the GFAP immunopositivity was more intense and therefore the trilaminar pattern was clearly recognizable, but it ceased in the dorsal septal nucleus (Figures 7j–l). Figures 7m–q demonstrate some of the abovementioned territories enlarged. The vessels were covered with wide, round end-feet (Figure 7m). Astrocytes were only seen in the corn snake septum (Figure 7q).

### Rest of the Diencephalon, Lizards

In gecko and monitor lizard, the GFAP-immunopositive elements were densely and near evenly distributed. Within the optic tract and chiasm, coarse processes were oriented parallel with the optic fibers.

**FIGURE 8:**
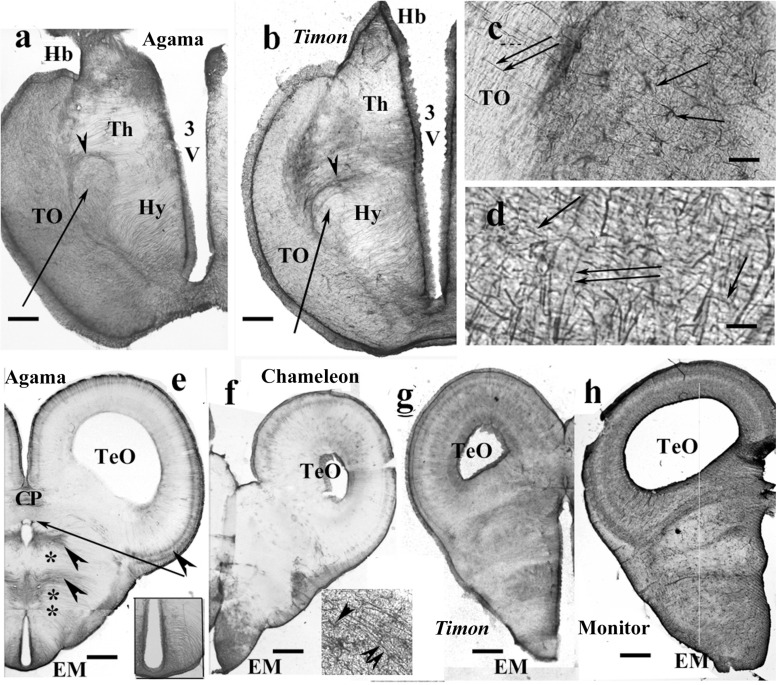
Lizards, diencephalon. 3V, third ventricle; CP, posterior commissure; EM, median eminence; FIV, interventricular foramen; GFAP, glial fibrillary acidic protein; Hb, habenula; Hy, hypothalamus; TeO, optic tectum; Th, thalamus; TO, optic tract. **(a)** Agama, behind the optic chiasm. The thalamus and hypothalamus are penetrated by radial processes, but the former one is relatively poor in GFAP. Arrow and arrowhead point to the lateral prosencephalic fascicle and the radial processes curved by it. Scale bar: 300 μm. **(b)** *Timon*, at a similar level. The glia formed by radial processes, GFAP-immunopositive zones alternate with GFAP-poor zones. Arrow and arrowhead point to the lateral prosencephalic fascicle and the radial processes curved by it. Scale bar: 250 μm. **(c)** Enlarged part of *Timon* diencephalon, the dense zone beside the optic tract with astrocytes (arrows). The radial glial processes penetrate the optic tract (double arrow). Scale bar: 80 μm. **(d)** *Timon*, enlarged detail of the optic tract. There are glial processes in parallel with the optic fibers (arrows) crossed by the radial glia (double arrow) Scale bar: 15 μm. **(e–h)** Cross sections at the posterior commissure. **(e)** Agama, the GFAP immunopositivity is confined to a few areas (arrowheads). Asterisk and double asterisk mark the GFAP-free areas of the thalamus and hypothalamus. The median eminence is penetrated by slightly arched glial processes (see enlarged in inset). The arrow points to the GFAP-free subcommissural organ. Scale bar: 550 μm. **(f)** Chameleon, almost free of GFAP except for the median eminence where astrocytes are also found. Inset: radial processes (double arrowhead) and astrocytes (arrowhead) in the median eminence. Scale bar: 400 μm. **(g,h)** *Timon* and monitor lizard had much more GFAP immunopositivity than the other two species. Scale bars: 450 and 600 μm.

In agama (Figure 8a), varied distributions of GFAP-immunopositive elements were found. The thalamus and hypothalamus were penetrated by radial processes, but the former one was relatively poor in GFAP. The forebrain bundles were revealed by their poor GFAP immunopositivity as they were only penetrated by sparse processes. The forebrain bundles curved the radial fibers pushing them apart. The epithalamus also contained coarse and dense ependymoglial fibers.

*Timon* had a similar structure (Figure 8b). In these species, zones of GFAP-immunopositive radial processes and zones free of GFAP alternated. The optic tract was separated with a dense zone containing astrocytes. The nucleus rotundus, triangularis, and ovalis were not recognizable in either agama or *Timon*.

In chameleon, the diencephalon was almost free of GFAP except for the optic tract and chiasma. The optic tract and chiasma were demarcated by a plexus of glial fibers which contained astrocytes (Figure 8c). The distal end of the radial fibers traversed the optic tract to reach the pial surface (Figures 8c,d).

At the posterior commissure, radial ependymoglial fibers formed alternating denser and less dense zones in agama (Figure 8e). GFAP immunopositivity was confined to these zones; otherwise, both thalamus and hypothalamus were free of GFAP immunopositivity. The median eminence was penetrated by slightly curved radial processes (Figure 8e inset). In chameleon, this area was very poor in GFAP immunopositivity (Figure 8f). The median eminence had only more GFAP immunopositivity and astrocytes intermingled with the radial glia.

The pretectum was intensely GFAP immunopositive in *Timon* (Figure 8g), gecko and monitor lizard (Figure 8h). The GFAP-immunopositive elements were near evenly distributed. The radial glial pattern was hardly recognizable.

### Mesencephalon, Lizards

**FIGURE 9:**
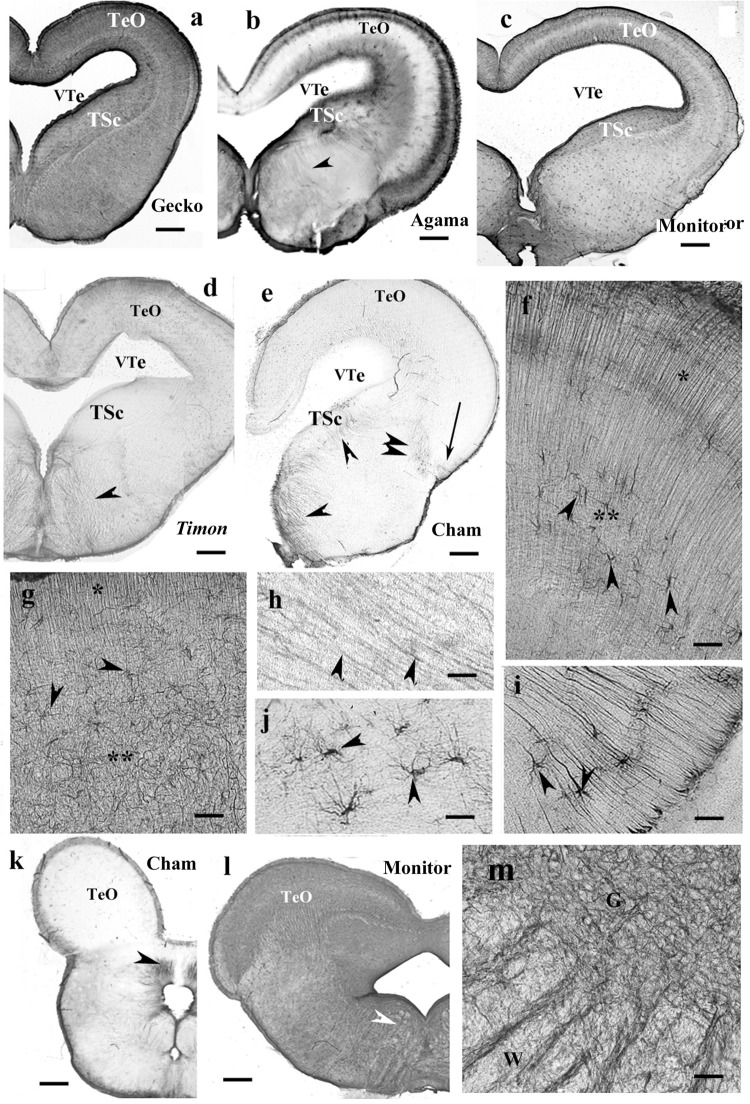
Lizard, mesencephalon (Cham, chameleon). G, W, gray and white matters; GFAP, glial fibrillary acidic protein; TeO, optic tectum; TSc, torus semicircularis; VTe, tectal ventricle. **(a)** Gecko, densely packed GFAP-immunopositive radial processes in the optic tectum. The tegmentum and torus semicircularis display a dense GFAP immunopositivity. Scale bar: 400 μm. **(b)** Agama, the GFAP immunopositivity is confined to the superficial and deep layers in the tectum. The torus and tegmentum are less intensely immunopositive than those in the gecko. The tegmentum has a loose system of radial processes (arrowhead, see enlarged in panel **h**). Scale bar: 1,500 μm. **(c)** Monitor lizard, also intensely GFAP-immunopositive with a lighter middle layer. Scale bar: 1,500 μm. **(d)** *Timon*, poor GFAP immunopositivity; relatively more is visible in the outer zone of the tectum and both sides of the midline in the tegmentum, where radial fibers are recognizable (arrowhead). Scale bar: 320 μm. **(e)** Chameleon, very poor in GFAP-immunopositive structures. There are a few groups of astrocytes (arrow, arrowheads). Scale bar: 320 μm. **(f)** A detail of the *Timon* optic tectum seen in panel **(d)**. The radial processes are recognizable in the superficial zone (asterisk) but less in the deep ones (double asterisk), where astrocyte-like figures are visible (arrowheads). Scale bar: 50 μm. **(g)** A detail of the torus semicircularis of gecko. Along the ventricle (asterisk) radial processes originate. Most of the territory (double asterisk) is occupied by irregular, sinuous processes. Arrowheads: astrocyte-like profiles. Scale bar: 50 μm. **(h)** Radial processes (arrowheads) enlarged from agama tegmentum. Scale bar: 10 μm. **(i)** Astrocytes from the chameleon; their position is pointed with a double arrowhead in panel **(e)**. Scale bar: 20 μm. **(j)** Chameleon, long processes from astrocytes (arrowheads). Their area is labeled with the arrow in panel **(e)**. Scale bar: 50 μm. **(k)** Chameleon, isthmus, the GFAP immunopositivity is confined (arrowhead). Scale bar: 450 μm. **(l)** Monitor lizard, isthmus, rich in GFAP immunopositivity. Arrowheads: the area enlarged in panel **(m)**. Scale bar: 1,500 μm. **(m)** Detail of the isthmus of the monitor lizard. G, W: gray and white matters Scale bar: 100 μm.

Tectum usually contained radial processes, but some interspecific differences were found. In gecko (Figure 9a), densely packed radial processes filled it. The density of side-processes, however, was uneven, which formed a weak layered pattern. In agama, the GFAP immunopositivity was confined to the superficial and deep layers of the tectum (Figure 9b). In the monitor lizard, the GFAP immunopositivity was almost complete, and a layered arrangement was recognizable (Figure 9c). *Timon* was rather poor in GFAP-immunopositive structures (Figure 9d) which confined to a subpial layer. The chameleon tectum was almost free of GFAP (Figure 9e). Astrocytes were found only in the *Timon* tectum (Figure 9f).

In most species, the ventricular surface of the torus semicircularis was rimmed by radial processes of ependymoglia, but in a short distance, they formed a plexus (Figure 9g). It was the one area where a few astrocyte-like profiles were seen in gecko (Figure 9g).

In the tegmentum, loose populations of radial processes were found in lizards (Figures 9a–c, enlarged in panel Figure 9h) with a denser irregular subpial zone along the ventral pial surface. In *Timon*, the radial processes grouped mainly at the midline (Figure 9d). In chameleon, the GFAP-immunopositive elements were only small groups of astrocytes and radial processes (Figures 9e,i,j).

In the isthmus, the chameleon also represented the weakest immunopositivity (Figure 9k), whereas gecko and monitor lizard the most intense ones (Figure 9l). In these latter two species, dense and looser glial systems distinguished the gray and white matters interdigitating with alternating strips into the intermediate zone between them (Figure 9m).

### Rest of the Diencephalon, Snakes

**FIGURE 10:**
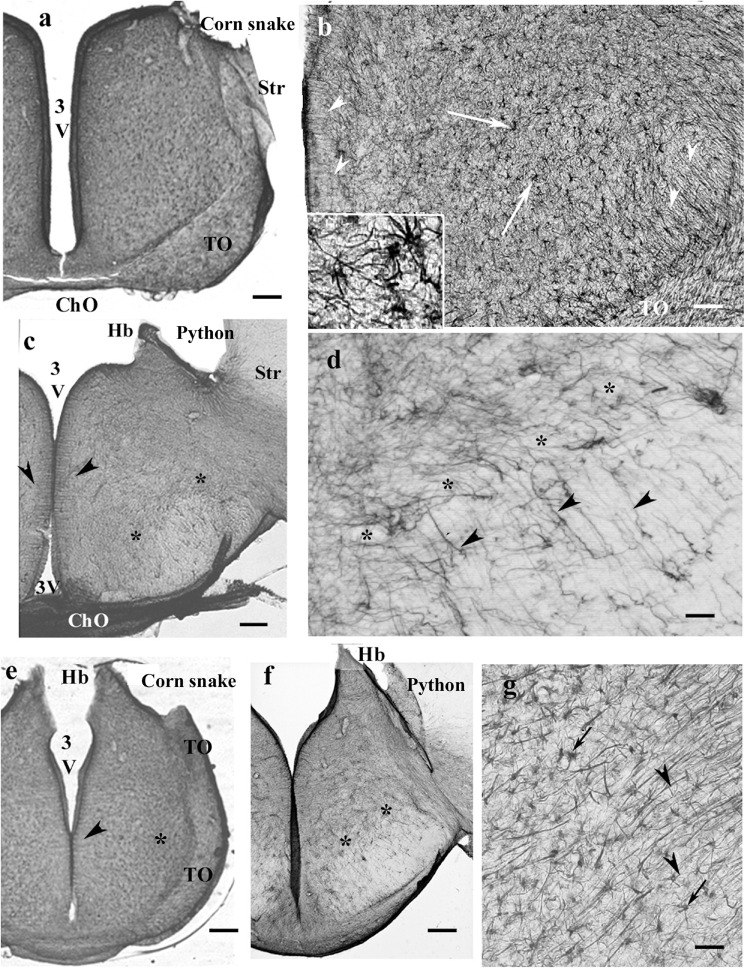
Snakes, diencephalon. 3V, third ventricle; ChO, optic chiasma; GFAP, glial fibrillary acidic protein; Hb, habenula; Str, striatum; TO, optic tract. **(a)** Corn snake, at the posterior edge of the optic chiasm. The whole diencephalon is GFAP-immunopositive. The striatum has been removed. Scale bar: 300 μm. **(b)** Enlarged part of corn snake diencephalon, the ependymal surface is to left. Arrowheads: radial glial processes, arrows: astrocytes; see enlarged in the inset. Scale bar: 80 μm. **(c)** Python at the optic chiasma. Ependymal origin of processes (arrowheads), complex plexus of tiny fibers (above the asterisks) and area of looser glial structure (below the asterisks). The striatum is free of GFAP immunopositivity. Scale bar: 300 μm. **(d)** Python diencephalon, the light area below the asterisks in panel **(c)** enlarged. Dense irregular (to left and up from the asterisks) and loose radially arranged processes (arrowheads). Scale bar: 50 μm. **(e)** Corn snake, posterior to the previous section; arrowheads: ependymal origin of radial glia, asterisk: a detail enlarged in panel **(g)**. Scale bar: 300 μm. **(f)** Python, posterior to the previous section; the marks are identical to those in panel **(c)**. Scale bar: 300 μm. **(g)** Corn snake, enlarged detail of panel **(e)**; arrows: astrocytes, arrowheads: radial processes. Scale bar: 40 μm.

GFAP-immunopositive elements occurred in every part. In corn snake, however, these elements were mainly astrocytes, although ependymal origins and subpial terminations of radial ependymoglia were also visible (Figures 10a,b). In python (Figures 10c,d) and boa, the glia was dense and less regular dorsally but loose and regular ventrally. Going caudalward, similar interspecific differences were found (Figures 10e–g).

**FIGURE 11:**
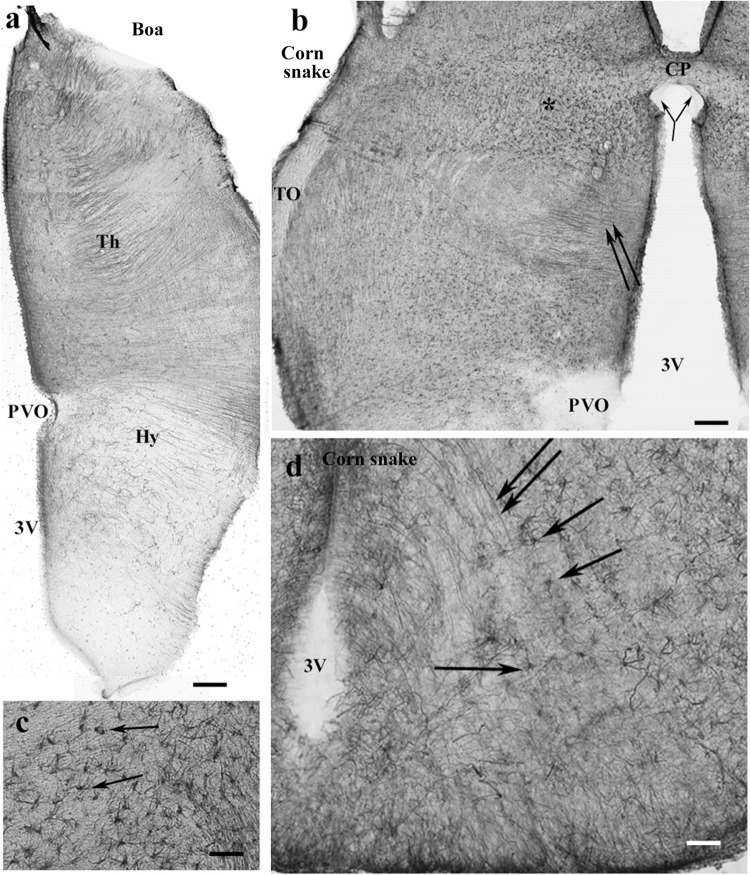
Snakes, pretectum. 3V, third ventricle; CP, posterior commissure; CPa, pallial commissure; DVR, dorsal ventricular ridge; GFAP, glial fibrillary acidic protein; Hy, hypothalamus; PVO, paraventricular organ; Th, thalamus. **(a)** Boa, the thalamus has a dense radial process system, whereas the hypothalamus has a loose one. The paraventricular organ is free of GFAP. Scale bar: 150 μm. **(b)** Corn snake, the dorsal part of the pretectum, asterisk marks the area with astrocytes enlarged in panel **(c)**; double arrow: radially oriented processes. The subcommissural organ (bifurcating arrow) is free of GFAP as well as the paraventricular organ. Scale bar: 120 μm. **(c)** Corn snake, enlarged detail around the asterisk in panel **(b)**, astrocytes (arrows). Scale bar: 50 μm. **(d)** Corn snake, radial processes (double arrow) and astrocytes (arrows) in the ventral part of the hypothalamus behind the median eminence. 3V, third ventricle. Scale bar: 30 μm.

In the pretectum in boa (Figure 11a) and python, the thalamus had a dense radial process system, whereas the hypothalamus had a looser one. The subcommissural and paraventricular organs were free of GFAP. In corn snake, the thalamus was also richer in GFAP than the hypothalamus but astrocytes predominated; few radial processes were only observed (Figures 11b,c). The median eminence was poor in GFAP but behind it, radial processes, and in corn snake, even numerous astrocytes were seen in the ventral part of the diencephalon (Figure 11d).

### Mesencephalon, Snakes

**FIGURE 12:**
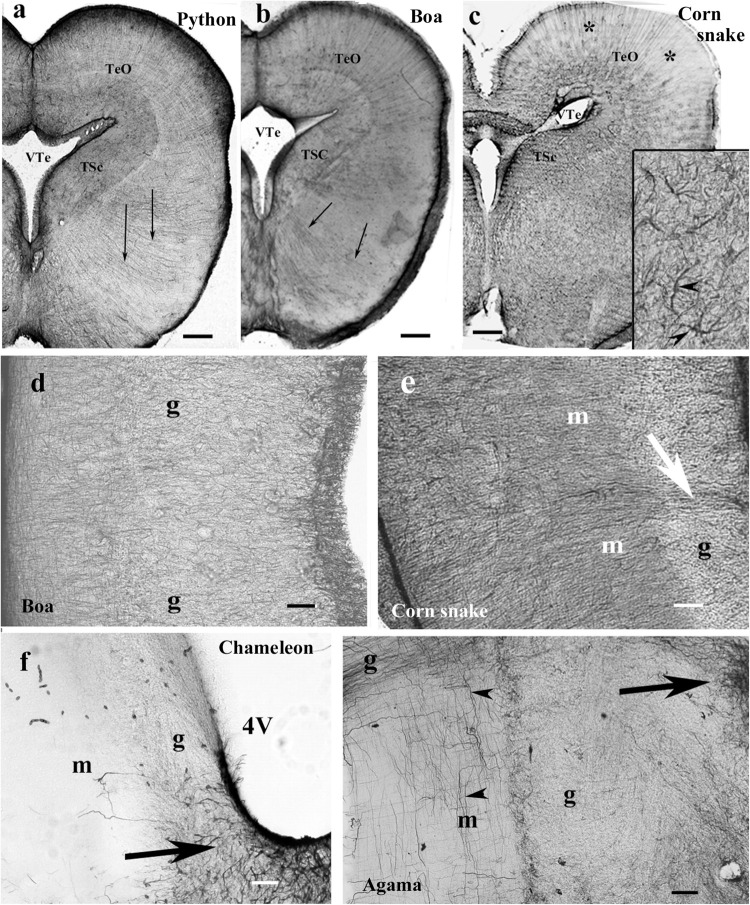
Mesencephalon (snakes) and cerebellum (snakes and lizards). 4V, fourth ventricle; g, m, granular and molecular layers of the cerebellum; GFAP, glial fibrillary acidic protein; TeO, optic tectum; TSc, torus semicircularis; VTe, tectal ventricle. **(a)** Boa, radial processes are found both in the optic tectum and in the tegmentum (arrows). **(b)** Python, similar glial structure like in boa. **(c)** Corn snake, note the less intense immunostaining in the superficial zone of the optic tectum (asterisks); except for this zone, astrocytes (see also inset, arrowheads) predominate everywhere. Scale bars: 250 μm. **(d–g)** Cerebellum. Three types were distinguished: **(d,e)**. Boa and corn snake. Dense Bergmann-like glial system, the molecular and granular layers are distinct mainly in the corn snake. In the corn snake, a large arrow points to the thick midline glial process system. Scale bar: 70 μm. **(f)** Chameleon. The cerebellum is almost free of GFAP-immunopositive processes, although the peduncle (arrow) is rich in them. The border between the molecular and granular layers is not recognizable. Scale bar: 100 μm. **(g)** Agama. The cerebellum is poor in GFAP-immunopositive perpendicular processes. A denser population is found only at the midline (arrow). The horizontal processes are therefore well recognizable (arrowheads). Scale bar: 70 μm.

In the mesencephalon of python (Figure 12a), radial processes span the full thickness of the brain wall in both the optic tectum and the tegmentum; a similar glial structure was found in boa (Figure 12b). The distribution of GFAP immunopositivity was more even than in the more rostral brain parts, except for a less intense immunostaining in the superficial zone of the optic tectum in corn snake (Figure 12c). In this species, no radial processes but astrocytes were found in the deeper part of the tectum, tegmentum (Figure 12c), and isthmus (not shown).

### Cerebellum, Lizards and Snakes

In the cerebellum, three types of astroglial systems were distinguished. (i) In boa, corn snake (Figures 12d,e), and python, a dense Bergmann-like glial system marked the molecular layer. Scarce horizontal glial processes crossed the Bergmann-like ones. In the midline, a thick glial bundle was found. (ii) In chameleon (Figure 12f), the cerebellum was almost free of GFAP-immunopositive processes, although they were abundant in the peduncle. The border between the molecular and granular layers was not recognizable. (iii) In agama (Figure 12g), the cerebellum was poor in GFAP-immunopositive processes but not free of them. Dense population was only found at the midline. Several horizontal processes were recognizable.

### Medulla and Spinal Cord, Lizards and Snakes

**FIGURE 13:**
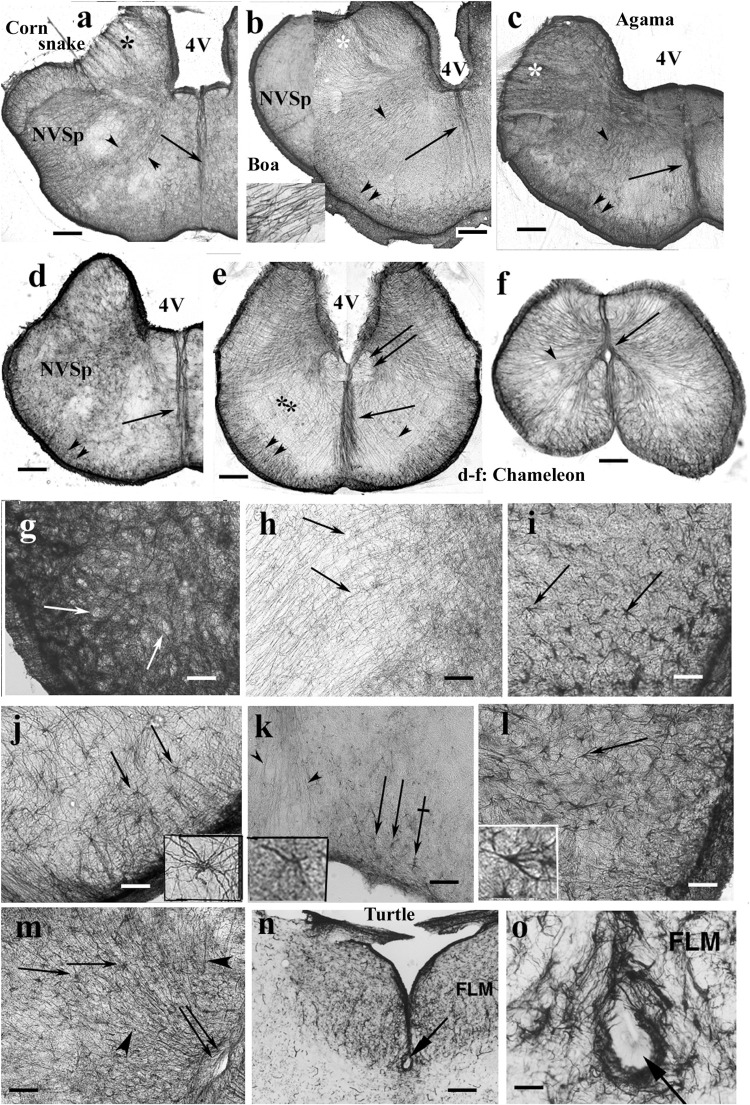
Rhombencephalon. 4V, fourth ventricle; FLM, medial longitudinal bundle; GFAP, glial fibrillary acidic protein; NVSp, nucleus of the spinal tract of trigeminal nerve. **(a–e)** Cross sections of rhombencephalon: **(a)** corn snake; **(b)** boa; **(c)** agama; **(d)** chameleon; **(e)** chameleon, more caudalward. Even chameleon is quite rich in GFAP in this brain part. Double arrow: medial longitudinal fasciculus. The basic pattern is radial glia (arrowheads). Along the pial surface, there is denser and non-radial glia (double arrowhead). Asterisks: emerges of cranial nerves (VII, VIII), note the interfascicular glial septa; arrow: midline glial bundle, inset: enlarged radial processes pointed by the arrowheads. Scale bar: 250 μm. **(f)** Chameleon spinal cord. Dense, mainly radial glia (arrowhead). Arrow: central canal. Scale bar: 200 μm. **(g)** Detail of the nucleus of the spinal trigeminal tract. Very dense system of glial processes; the light holes (arrows) correspond to neurons. Scale bar: 50 μm. **(h)** Dense, less regular and loose, radial glia (arrows) populations. Enlarged detail labeled with double asterisk in panel **(e)**. Scale bar: 50 μm. **(i)** *Timon*, astrocytes in the brain stem (arrows). Scale bar: 50 μm. **(j)** Python, astrocytes (arrows) with unusually long processes in the brain stem; see a cell enlarged in the inset. Scale bar: 50 μm. **(k)** Corn snake, astrocytes (arrows) in the ventromedial part of the brain stem; crossed arrow points to a cell enlarged in the inset. Arrowheads: processes in the midline glial bundle. Scale bar: 100 μm. **(l)** Corn snake, astrocytes in the ventrolateral part of the brain stem; arrow points to a cell enlarged in the inset. Scale bar: 50 μm. **(m)** A detail of the chameleon spinal cord. Radial glia (arrowheads) and astrocytes (arrows); double arrow: central canal. Scale bar: 100 μm. **(n,o)** The subtrochlear organ (arrow) in the turtle *(Trachemys scripta elegans*) brain stem. Scale bars: 150 and 30 μm.

The medulla was rich in GFAP-immunopositive elements in each species even in the chameleon (Figures 13a–e). The radial pattern was usually recognizable. Glial processes formed a thick septum in the midline. Along the ventral pial surface, a dense, less regular glial population was found. The medial longitudinal fasciculus was recognizable (Figure 13e), as well as the roots of the large nerves (Figures 13a–d). The spinal cord showed a similar dense radial glia, even in chameleon (Figure 13f).

Most nuclei of the brain stem were not recognizable in GFAP-immunostained sections. Neither nucleus magnocellularis cochlearis nor nucleus laminaris was recognizable in contrast to caiman (see also Kálmán and Pritz, 2001). A very dense system of glial processes marked the nucleus of the spinal tract of the trigeminal nerve (Figure 13g), but the density was uneven (Figure 13h). Astrocytes were found in several species, e.g., python, corn snake, *Timon*, and chameleon (Figures 13i–m). Subtrochlear organ was not found in Squamata, in contrast to the turtle brain stem (Figures 13n,o). The results are semiquantitatively summarized in a Supplementary Table.

### Brief Description of the Turtle and Caiman Brains

Turtle brains showed astroglial architectures similar to those described in our former paper (Kálmán et al., 1994); therefore, they have not been described here in detail. The main points are that neither extended GFAP-poor areas nor astrocytes were found. The caiman astroglial architecture has been described in *Caiman crocodilus* (Kálmán and Pritz, 2001); therefore, here, the few differences found in *Paleosuchus* are only mentioned. The density of radial processes did not distinguish the striatum and the DVR. The radial glia was interwoven with fine non-radial processes so densely that the general impression indicated a three-dimensional network rather than a unidirectional system. The system of especially thick radial astroglial processes described in the DVR of *C. crocodilus* was not found in *Paleosuchus*.

## Discussion

The main results can be summarized in four statements:

The original radial ependymoglia are enmeshed by secondary, non-radial processes beyond recognition in several brain areas—a similarity to other reptiles.

Astrocytes occur but usually as complements of the radial glia—a similarity to caiman but not to turtles.

In most species, extended areas are poor in GFAP or avoid it.

The astroglial pattern shows a great diversity in different Squamata species—these latter two features were not found in other reptiles.

### The Radial Glia and Their Modifications

The astroglia of reptiles is described as radial ependymoglia (Achúcarro, 1915; King, 1966; Monzon-Mayor et al., 1990; Yanes et al., 1990; Lazzari and Franceschini, 2001, 2005a,b). In fact, in several brain areas, the radial system is not recognizable either in Squamata, or in turtles, or crocodilians (see also Kálmán et al., 1994; Kálmán and Pritz, 2001). The radial pattern is enmeshed beyond recognition with non-radial processes. The ependymal origin is also not recognizable at each process. The fact that the original system is radial ependymoglia has only been revealed by developmental studies (Monzon-Mayor et al., 1990; Yanes et al., 1990; Kálmán et al., 1997).

The glial network forms local modifications. A characteristic one is the trilaminar structure of pallium which has also been shown in *Anolis sagrei*, *Eublepharis maculata*, *Podarcis sicula* (Lazzari and Franceschini, 2001, 2005a,b), *Gallotia galloti* (Font et al., 2001), and *Tarentola mauritanica* (Ahboucha et al., 2003) but without explanation. In the middle layer of neurons has a looser system of astroglial processes than in the upper and deeper zones. No similar adaptation is found in turtles and caiman, in which the pallium is evenly densely rich in GFAP (see also Kálmán et al., 1994; Kálmán and Pritz, 2001).

Brain nuclei are unfrequently revealed by GFAP immunohistochemistry. The nuclei laminaris and cochlearis were identified in caiman (Kálmán and Pritz, 2001) but not in snakes, lizards, and turtles (see also Kálmán et al., 1994). The reason is probably that vocal signals are important in Crocodilia but not in the other reptiles.

Some brain tracts appear “light” due to the loose arrangement of astroglial elements among the myelinated axon bundles. It was also found in other reptiles (Kálmán et al., 1994; Kálmán and Pritz, 2001). On the other hand, the checker-table alternation of denser and looser astroglia, which was characteristic of the reticular formations of turtle and caiman (Kálmán et al., 1994; Kálmán and Pritz, 2001) was not found in Squamata except for a small area in monitor lizard; we suppose that the reason is a finer mixing of neuron groups and neuropil.

The paraventricular and subcommissural organs also proved to be free of GFAP immunopositivity in chicken (Kálmán et al., 1993; Goren et al., 2006) and mammals (Didier et al., 1986; Hajós and Kálmán, 1989; Goren et al., 2006; note that the paraventricular organ does not exist in adult mammals, Tsuneki, 1986). These organs are formed by thick ependyma (Röhlich and Vígh, 1967), which may underlie their GFAP immunonegativity. The subtrochlear organ has a similar structure according to Uryu et al. (1988) and proved to be GFAP-immunonegative in chicken (Goren et al., 2006).

### Astrocytes

Since astrocytes are the predominant astroglial elements in the bird and mammalian brains (King, 1966; Achúcarro, 1915; Appel, 2013), they are to be regarded as an apomorphic feature in lizards, too. Astrocytes have been demonstrated at least in some areas in several species (Dactyloidae: *Anolis carolinensis* cerebellum, Dahl et al., 1985; *Anolis sagrei* mesencephalon and medulla, Lazzari and Franceschini, 2005a; Lacertidae: *Gallotia galloti* mesencephalon, Yanes et al., 1990; *Lacerta lepida*, recently *Timon lepidus*, Bodega et al., 1990; *Podarcis sicula*: optic tectum and spinal cord, Lazzari and Franceschini, 2001; Geckonidae: *Eublepharis macularius*: diencephalon, mesencephalon, and medulla, Lazzari and Franceschini, 2005b; Scincidae: *Eumeces algeriensis*, Ahboucha et al., 2003). The telencephalon was usually found free of astrocytes: *Podarcis sicula* (Lazzari and Franceschini, 2001), *Gallotia galloti* (Yanes et al., 1990), *Eublepharis macularius* (Lazzari and Franceschini, 2005b), maybe except for the *Anolis sagrei* (Lazzari and Franceschini, 2005a). Only one comment refers to snake (*Elaphe*—recently*: Pantherophis quadrivirgata* hippocampus, Onteniente et al., 1983). It is noteworthy that the present study also found astrocytes in different numbers and localization in the different species. In gecko, they were almost absent, whereas in corn snake, in the diencephalon and mesencephalon, their density matched or even surpassed that of tanycytes.

The absence of astrocytes in the turtles investigated corresponds to that found formerly in *Pseudemys* (recently: *Trachemys*) *scripta elegans* (Kálmán et al., 1994) and *Mauremys leprosa* (Kálmán et al., 1997). Independent studies with GFAP immunostaining also did not detect astrocytes (*Clemmys japonica*: Onteniente et al., 1983; *Pseudemys scripta elegans*: Dahl et al., 1985; Kriegstein et al., 1986; *Trionyx sinensis*: Lazzari and Franceschini, 2006). In *Caiman*, astrocytes occurred at several places but nowhere predominated (see also Kálmán and Pritz, 2001).

The mixed populations of radial glia and astrocytes in Squamata (as well as in caiman, Kálmán and Pritz, 2001) suggest that the appearance of astrocytes had preceded and maybe promoted rather than followed the increase of brain size and complexity, which took place in birds and mammals. The mixed occurrence of astrocytes and radial glia modifies Reichenbach’s supposal (Reichenbach et al., 1987) that the astrocytes formed to replace radial glia, which was “over-elongated” due to the thickening of the brain wall during evolution. In Mugnaini (1986), “a division of labor may be required……a separation of parietal functions (ependyma) and intrinsic or centralized functions (astrocytes).” In radial glia, single nuclei control long cells spanning the entire brain wall, which is not the case with astrocytes. They form a versatile network and provide local adaptability. At first, they completed rather than replaced radial glia.

### Glial Fibrillary Acidic Protein-Free Areas

Mammalian (rat, Kálmán and Hajós, 1989; Zilles et al., 1991) and avian (chicken, Kálmán et al., 1993; quail, Cameron-Curry et al., 1991) brains contain extended areas almost free of GFAP immunopositivity, what is not found in the brains of crocodilians and turtles. Avian homologs of GFAP-rich turtle or *Caiman* brain areas were frequently almost free of GFAP (e.g., the molecular layer of the cerebellum, the superficial layers of the tectum, most of DVR, Kálmán et al., 1993, 1994; Kálmán and Pritz, 2001). Therefore, the withdrawal of GFAP immunopositivity may be regarded as an apomorphic feature (Kálmán, 2002).

No former study emphasized the occurrence of large GFAP-poor or -free brain areas in Squamata. The absence of such areas in the turtles and caiman is in accordance to that we formerly found in *Trachemys scripta elegans* (Kálmán et al., 1994), *Mauremys leprosa* (Kálmán et al., 1997), and *Caiman crocodilus* (Kálmán and Pritz, 2001). Independent studies with GFAP immunostaining also did not detect GFAP-free areas (*Clemmys japonica*, Onteniente et al., 1983; *Trachemys scripta elegans*, Dahl et al., 1985; Kriegstein et al., 1986; *Trionyx sinensis*, Lazzari and Franceschini, 2006).

In several species investigated (agama, chameleon, *Timon*, snakes), the extension of GFAP-free areas matches that found in birds. However, there are differences in the homologous areas: the upper layers of the tectum and in some cases the molecular layer of the cerebellum contained GFAP in Squamata but not in birds (Linser, 1985; Roeling and Feirabend, 1988; Kálmán et al., 1993). In Squamata, there is no GFAP-immunopositive area corresponding to the ectostriatum (present terminology: endopallium, Reiner et al., 2004) of birds.

The lack of GFAP expression in a brain area does not mean the lack of astroglia. Astrocytes express GFAP at different levels, and the GFAP levels of brain areas can be different (Ludwin et al., 1976; Connor and Berkowitz, 1985; Linser, 1985; Patel et al., 1985; Hajós and Kálmán, 1989; Hajós and Zilles, 1995). GFAP seems to be involved in several features of astrocytes. Walz (2000) distinguished two electrophysiologically different astrocyte types: one (“passive”) is rich in GFAP and has strong K+ accumulation, and the other (“complex”) is poor in GFAP but has intense Na-K currents. GFAP influences the swelling of astrocytes (Ding et al., 1998; Bélanger et al., 2002; Anderova et al., 2014). Glutamate/glutamine transformation appears to correlate inversely with the GFAP content (Patel et al., 1985; Didier et al., 1986; Ong et al., 1993; Pekny et al., 1999). The stiffness of glial processes correlates with intermediate filament expression (Galou et al., 1997; Lu et al., 2011) as well as the composition of extracellular matrix produced by astrocytes (Menet et al., 2001).

The increased expression of GFAP may impair the synaptic plasticity (Missler et al., 1994; Finch, 2003). These data, together with the absence of GFAP in the DVR in bird (chicken, Kálmán et al., 1993) and in the cortical layers 2-3-4 in mammal (rat, Ludwin et al., 1976; Kálmán and Hajós, 1989; Zilles et al., 1991), raise the possibility that the GFAP-free areas expand during evolution, which may promote the increase of brain plasticity (Kálmán, 2002). The present observations are in accordance with this.

### Diversity and Evolution

The astroglial architecture of Squamata represents a wide spectrum. No former study emphasized great interfamily diversity in Squamata. Only Ahboucha et al. (2003) described differences in the astroglial architectures of a gecko (*Tarentola mauritanica*, it represents another family, Geckonidae than our *Eublepharus macularius*), an agama (*Agama impalearis*), and a skink (*Eumeces algeriensis*).

In contrast to Squamata, no interfamily differences were found between turtles (including the Pleurodira *Pelomedusa*) and crocodilians. It may be attributed to that Squamata is a relatively young group with such an intense diversity. Note that most of the extant reptile species belong to this group. The data (see above) also indicate that the Squamata brains are rather apomorphic related to the other reptilian brains. It may correlate with that Squamata display quite complex behavioral phenomena related to other reptiles (Zug et al., 2001).

It is to be emphasized that both astrocytes and GFAP-poor areas evolved during separate evolutions in mammals, birds, and squamates, since they belong to different clades (Synapsida, Diapsida-Archosauria, and Diapsida-Lepidosauria), and these features are found neither in crocodilians, the closest extant relatives of birds, nor in turtles, which probably represent the most ancient astroglial architecture in Amniotes (Kálmán, 2002). Whether turtles are anapsid parareptiles (Carroll, 1988) or they are diapsids and form a sister group of lepidosaurs, or archosaurs, or all the other diapsids (for a survey, see Iwabe et al., 2005), it is beyond the scope of our study; for recent opinions, see Lyson et al. (2012), Joyce (2015), Adams et al. (2016), or Shaffer et al. (2017). In either case, the turtles are more ancient than Squamata (Joyce, 2007; Lichtig et al., 2018).

**FIGURE 14:**
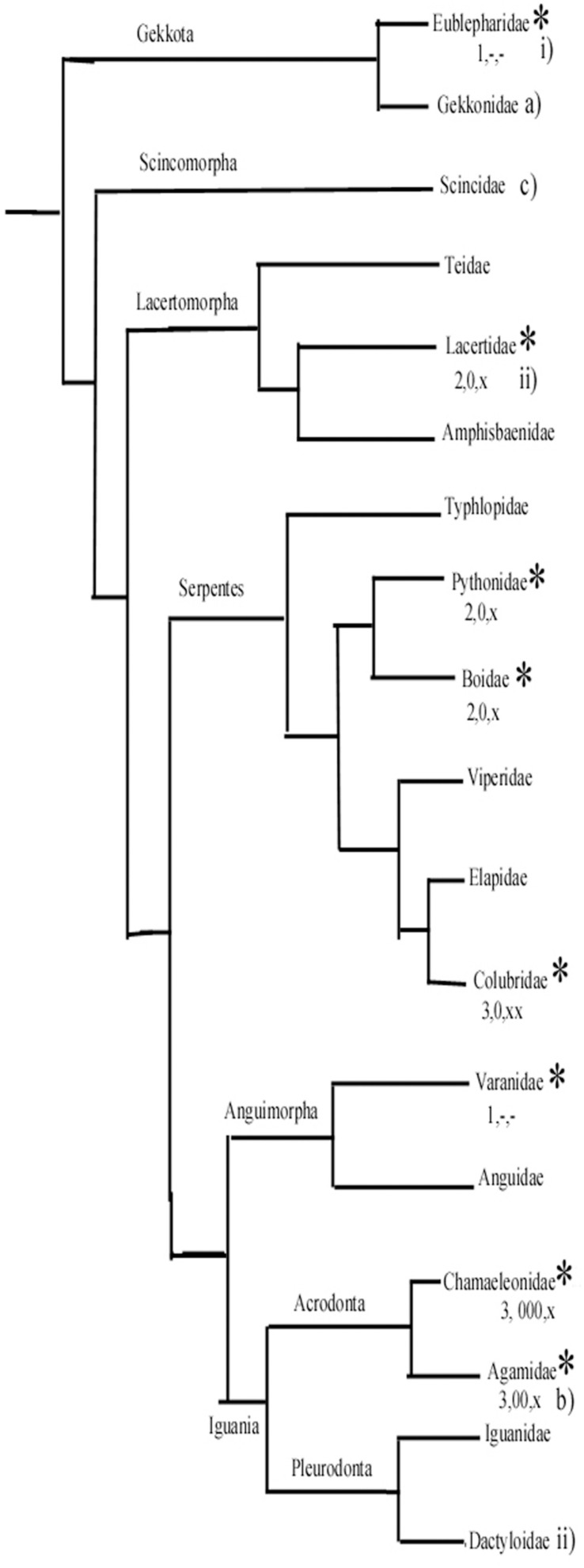
A simplified phylogenetic tree of Squamata. It has been constructed based on Wiens et al. (2012) and Pyron et al. (2013). It demonstrates the positions of groups whose representatives were studied by us (asterisks) and a few other important groups. (1–3) From our study: (1) Ancient-type plesiomorph glial structure according to our study. (2) Intermediate. (3) Most apomorphic glial structure. Astrocytes: –, none or minimal; x, in some confined areas; xx, predominant in large areas. Glial fibrillary acidic protein (GFAP)-poor or free areas: –, none; 0, medium; 00, 000, predominant. **(a–c)** Ancient, intermediate, and most apomorphic according to Ahboucha et al. (2003). i, ii, less and more advanced according to Lazzari and Franceschini (2001, 2005a, 2005b). (The Dactyloidae group is represented by *Anolis sagrei* Lazzari and Franceschini, 2005a).

Surveying the relations of the families within Squamata (Figure 14), Ahboucha et al. (2003) estimated that the astroglial architecture of *Eumeces algeriensis*, Scincoidae is apomorphic, since astrocytes are abundant; *Tarentola mauritanica*, Geckonidae represents a relatively ancient state, and *Agama impalearis* has an intermediate degree between them (Figure 14). Lazzari and Franceschini (2001, 2005a,b) supposed that the gecko *Eublepharius macularius*, which had a relatively extended vimentin immunopositivity, represents a more ancient form to the *Podarcis sicula* (Lacertidae) and the *Anolis sagrei* (Dactyloidae) brains.

According to our results, in gecko and monitor lizard, the astroglia have plesiomorph features similar to those found in turtles. The geckos belong to a sister group of all other Squamata (Wiens et al., 2012; Pyron et al., 2013). On the other hand, agama and chameleon, which have the most extended GFAP-free areas and have astrocytes in some brain areas, belong to the Iguania, which is considered to be an apomorphic group opposite Sacoglossa, which comprises all the other Squamata. The monitor lizards, like geckos, have plesiomorph astroglial features. Their clade separated from snakes later, however, then from the clade containing lacertids (Wiens et al., 2012; Pyron et al., 2013). Therefore, we suppose that the apomorphic features of astroglia evolved independently, in parallel evolution in lacertids and snakes. Of snakes, the richest astrocytic system was found in corn snake, incomparably surpassing that of boa and python.

## Conclusion

The astroglial structure of Squamata seems to be the most apomorphic one among reptiles. Their astroglial system shows a high interspecific diversity, in several species, there are large GFAP-free areas and astrocytes. These features of the Squamata glial system developed independently from that of birds and mammals. There was no systematic difference between the glial structure of snakes and lizards. The differences of GFAP contents of identical brain areas in different species may promote understanding of the role of GFAP. Our results suggest that brain evolution in the young group of Squamata is still an intense phase. Our findings are in accordance with the supposal based on our previous studies that the GFAP-free areas expand during evolution.

## Data Availability Statement

Requests to access the datasets should be directed to MK, kalmanprof@gmail.com.

## Ethics Statement

The animal study was reviewed and approved by Committee on the Care and Use of Laboratory Animals of the Council on Animal Care at the Semmelweis University of Budapest, Hungary (22.1/3491/003/2008), the permission of Hungarian authorities (KA-1928, dated from May 31, 1916).

## Author Contributions

DL contributed to the perfusions and histology. MK contributed to the histology and manuscript. Both the authors contributed to the article and approved the submitted version.

## Conflict of Interest

The authors declare that the research was conducted in the absence of any commercial or financial relationships that could be construed as a potential conflict of interest.
