# Supplementary material for: Distribution of GFAP in Squamata: Extended Immunonegative Areas, Astrocytes, High Diversity, and Their Bearing on Evolution
Source: Front Neuroanat. 2020 Aug 14;14:49. doi: 10.3389/fnana.2020.00049 (PMC7457009; doi:10.3389/fnana.2020.00049)
Supplement: Supplementary file 1 [file Data_Sheet_1.PDF]

Supplement Table –

A semiquantitative summary of results

	Med.	Dors.	Lat.	DVR	Striatum	Thalamus	Hypothal.	Pretect	Tectum	Tegm.	Mdl	Cbl
Gecko	II	II	#	##	II	##/II	##/II	II/II	II	II#	II	
Monitor	II	II	#	##	II	##/II	##/II	II/I	II	II	II	
Timon	II	II	0/II	0/##	0/##	II/Ix/0	II/I/0	II/I	II/0x	II/I	IIx	
Agama	II	0/II	0	0	Ix	II/I	II/I	II/0	II/0	II/I	IIx	
Cham.	II/0	0	0	I	I	0(I)(x)	0(I)(x)	0(I)(x)	0(I)	0(I)(x)	IIx	
Boa	0/II	0/II	0/II	0	0/##	##	##/I	II/I	II	II	IIx	
Python	0/II	0/II	0/II	0	0/##	##	##/I,	II/I	II	II	IIx	
Cornsn.	0/II	0/II	0/II	0/#	0/##	xxI	##/xI	xxI	IIx	xxI	IIx	

Abbreviations:

Cbl – cerebellum; Cham – chameleon; Cornsn- cornsnake; Med, Dors.,Llat. – the subdivisions of pallium; Mdl – medulla;

Pretect –pretectum; Tegm. – the tegmentum of mesencephalon.

Symbols:

0 – GFAP-free

(I)–confined radial glia groups, I - less dense radial glia, II - dense radial glia

(#)- confined groups; #,## - less dense or dense glial processes, radial system is not or hardly recognizable

(x) –confined small astrocyte groups, x – astrocytes, xx – dense astrocyte population

Symbols together (e.g. IIx) – mixed populations,predominant first; with zero (e.g. 0I) - scarce populations

Symbols separated (e.g. II/x) – different glial populations in the different areas of the brain part
